# Run length distribution of dimerized kinesin-3 molecular motors: comparison with dimeric kinesin-1

**DOI:** 10.1038/s41598-019-53550-2

**Published:** 2019-11-18

**Authors:** Si-Kao Guo, Xiao-Xuan Shi, Peng-Ye Wang, Ping Xie

**Affiliations:** Key Laboratory of Soft Matter Physics, Institute of Physics, Chinese Academy of Science, Beijing, 100190 China

**Keywords:** Biophysics, Computational biophysics

## Abstract

Kinesin-3 and kinesin-1 molecular motors are two families of the kinesin superfamily. It has been experimentally revealed that in monomeric state kinesin-3 is inactive in motility and cargo-mediated dimerization results in superprocessive motion, with an average run length being more than 10-fold longer than that of kinesin-1. In contrast to kinesin-1 showing normally single-exponential distribution of run lengths, dimerized kinesin-3 shows puzzlingly Gaussian distribution of run lengths. Here, based on our proposed model, we studied computationally the dynamics of kinesin-3 and compared with that of kinesin-1, explaining quantitatively the available experimental data and revealing the origin of superprocessivity and Gaussian run length distribution of kinesin-3. Moreover, predicted results are provided on ATP-concentration dependence of run length distribution and force dependence of mean run length and dissociation rate of kinesin-3.

## Introduction

Kinesins are a large superfamily of motor proteins capable of moving on microtubule (MT) filaments by hydrolyzing ATP^1–3^. They are responsible for a variety of biological functions in cell such as intracellular cargo transport, chromosome segregation, spindle assembly, cytoskeletal organization, etc.^3^. Among the kinesin superfamily, kinesin-3 motors constitute one of the largest families^4,5^, which are involved in functions of vesicle transport^6–12^, signaling^13,14^, mitosis^15,16^, interkinetic nuclear migration in neural stem cells^17^, transport of alphaherpesvirus particles in neurons^18,19^, early embryonic development^20^, and so on.

While a lot of researches have focused on studies of dynamics of kinesin-1 motors, the dynamics of kinesin-3 motors has also been studied insightfully^5,21,22^. Recently, using Forster resonance energy transfer microscopy in live cells, Soppina *et al*.^23^ showed that while in monomeric state kinesin-3 is inactive in motility, the dimerization results in superprocessive motion, with an average run length of ~ 10 μm, which is more than 10-fold longer than that of kinesin-1. Considering that under no load the kinesin dimer moves on average with a constant velocity and during the processive movement there is a constant rate of dissociation from MT, a single-exponential or nearly single-exponential distribution of run lengths would be expected^24,25^, as the available experimental data showed for kinesin-1^23,26,27^, kinesin-4 KIF21B^28^, kinesin-7 CENP-E^29^, kinesin-12 Kif15^30^, and so on. However, it was found surprisingly that the dimerized kinesin-3 has approximately a Gaussian-form distribution of run lengths, which is deviated significantly from the single-exponential form^23^. Furthermore, Scarabelli *et al*.^31^ found that substitutions of prominent kinesin-3 residues that contribute to interaction energy with MT decrease kinesin-3 processivity by about 10-fold, thus approaching kinesin-1 levels. However, while the family-specific K-loop provides kinesin-3 motors with a high MT affinity in ADP state of the motor, the mutation of the K-loop has little effect on the superprocessive motion of the dimeric motors^32^.

Despite the extensive studies, the mechanism of how kinesin-3 dimers show superprocessivity is unclear. Is the higher MT affinity sufficient to give the superprocessivity of kinesin-3 dimers? While the K-loop enhances the MT affinity, why do mutations of the K-loop have little effect on the superprocessivity of kinesin-3 dimers? More puzzlingly, why do kinesin-3 dimers have approximately a Gaussian form of run length distributions rather than a nearly single-exponential form? The purpose of this work is to address these unclear issues, which is critical to the molecular mechanism of the mechanochemical coupling of kinesin-3 dimers.

## Methods

### The model of processive movement of kinesin-1 and kinesin-3 dimers and simulation methods

**Figure 1:**
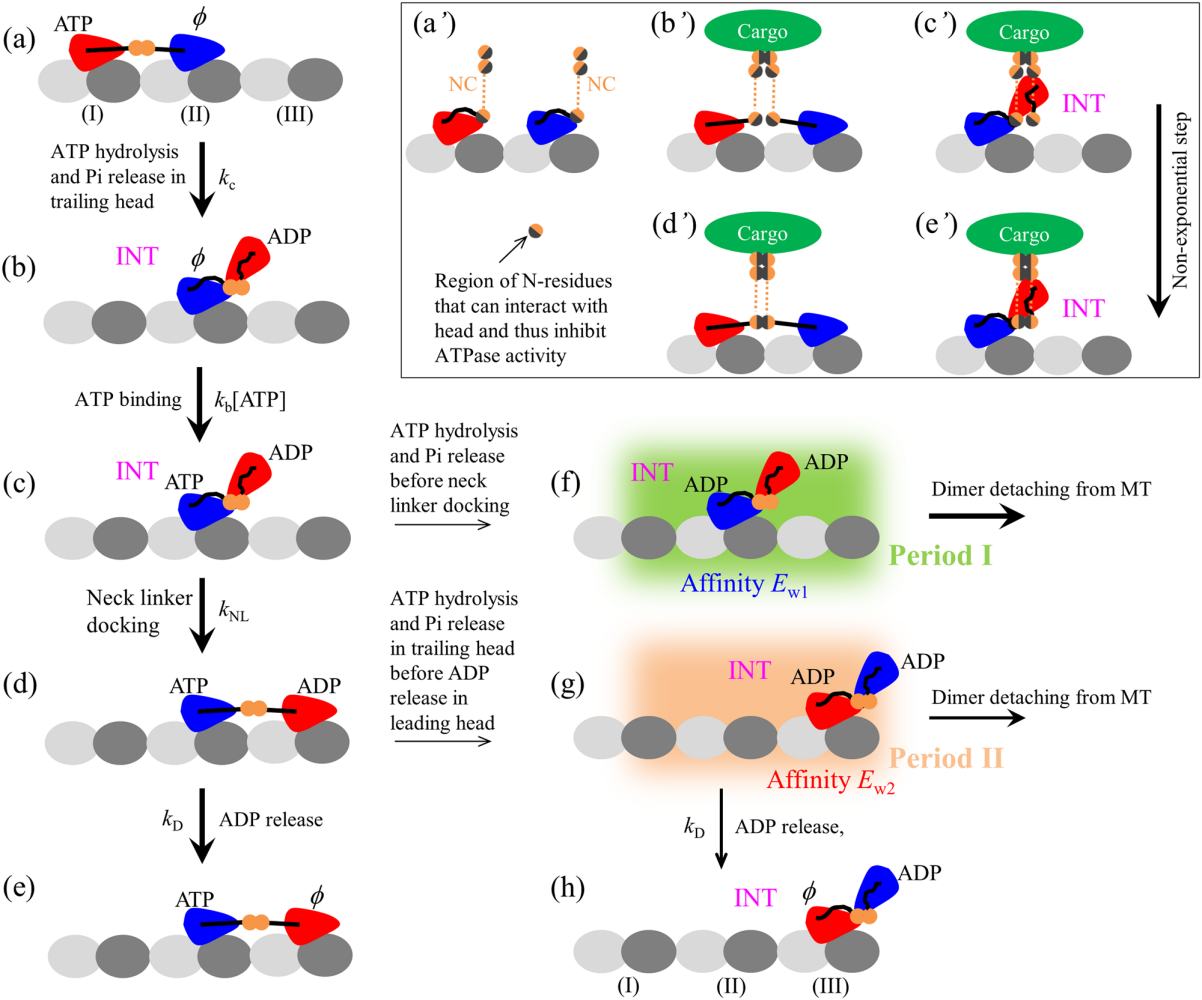
Schematic of a typical forward stepping of dimeric kinesin-1 and kinesin-3. (**a**) The trailing head in ATP state binds strongly to binding site I on MT while the leading head in nucleotide-free (*ϕ*) state binds strongly to site II. The rate of ATP hydrolysis and Pi release of the trailing head with the forward orientation of the NL is much higher than that of the leading head without the forward orientation of the NL. **(b)** After ATP hydrolysis and Pi release in the trailing head, due to the very weak affinity (*E*_w1_) between the ADP-head and the local site I, the ADP-head detaches easily from site I and diffuses rapidly to the INT position relative to the MT-bound head, where the two heads have a high affinity. **(c)** ATP binds to the MT-bound *ϕ*-head. **(d)** The NL docking of MT-bound head takes place, weakening the interaction between the two heads, and the tethered ADP-head then diffuses rapidly to the forward site III on MT. Note that the NL docking provides an energy barrier *E*_NL_ to prevent the tethered ADP-head from moving backward. **(e)** Stimulated by MT, ADP is released from the leading head. State in (e) is the same as that in (a) except a forward step was made. **(f)** From (c), ATP hydrolysis and Pi release can also occur occasionally in the MT-bound head before its NL docking. Before the affinity of the MT-bound ADP-head for the local site II changes from *E*_w1_ to *E*_w2_ (called Period I), the dimer can detach easily from MT by overcoming the very weak affinity *E*_w1_. **(g)** From (d), ATP hydrolysis and Pi release can also occur occasionally in the trailing head before ADP is released from the leading head. The dimer also has a large probability to detach from MT before ADP is released from the MT-bound head (called Period II) by overcoming weak affinity *E*_w2_. **(h)** From (**g**), the dimer has not detached from MT until ADP is released from the MT-bound head. State in (**h**) is the same as that in **(b**) except a forward step was made. Inset (a’ – e’) is the schematic illustration of the intermolecular interaction between two NC segments and intramolecular interaction of N-residues with the head during cargo-mediated dimerization of kinesin-3. **(a’)** Before cargo-mediated dimerization, the intramolecular interaction of the N-residues with head inhibits its ATPase activity. **(b’**,**c’)** After cargo binding, firstly the C-terminal residue (adjacent to the cargo) in one NC helix forms an intermolecular interaction with the corresponding residue in another NC helix. Then, the intermolecular interactions between residues in the two helixes form successively. Before formation of the intermolecular interaction between N-residues in the two helixes, in the state with two heads bound to MT, the stretching of the two NLs causes the N-residues to be away from their heads, with no intramolecular interaction between the N-residues and head. The two heads thus have the ATPase activity (b’). In INT state, the N-residues are close to the head and the intramolecular interaction between them is present, inhibiting the ATPase activity of the MT-bound head (c’). **(d’, e’)** After complete formation of the coiled-coil between two NC helixes, the formation of the intermolecular interaction between N-residues in the two helixes prevents the intramolecular interaction between the N-residues and MT-bound head in both the state with the two heads bound to MT (d’) and INT state (e’). Thus, in both states the MT-bound head has the ATPase activity.

The model for kinesin-1 has been proposed before^33,34^, based on which the available single-molecule experimental data on the force dependence of the mean velocity and run length of the wild-type kinesin-1 and the mutant one with extension of its two neck linkers^27,35^ were reproduced quantitatively. In particular, the puzzlingly dramatic asymmetry of the mean run length of kinesin-1 dimers with respect to the direction of external load acting on the coiled-coil stalk was explained well^33,34^. Here, we consider that kinesin-3 and kinesin-1 dimers share the same mechanism of processive movement on MT. The model for the pathway of a typical forward stepping and dissociation from MT of kinesin-3 and kinesin-1 dimers is schematically shown in Fig. 1, which is built up based mainly on the following pieces of experimental and computational evidence and/or arguments.

(i) A kinesin head in nucleotide-free (*ϕ*), ATP or ADP.Pi state has a high binding affinity to MT, whereas in ADP state has a low affinity (denoted by *E*_w2_), as available experimental data showed^36–40^. Moreover, it is argued that upon Pi release the binding energy of the ADP-head with the local binding site on MT (denoted by *E*_w1_) is temporarily weaker than that with other unperturbed binding sites (i.e., *E*_w1_ < *E*_w2_)^41–43^, which is supported by recent all-atom molecular dynamics (MD) simulations^44^. This can be understood as follows. The large conformational changes in the local MT-tubulin heterodimer induced by the strong interaction with *ϕ*-, ATP- or ADP.Pi-head^45^ result in the local tubulin having a further weaker interaction with the ADP-head than other unperturbed tubulins. In a time of *t*_r_ the local tubulin relaxes to its normally unperturbed conformation, with the interaction energy of the local tubulin with the ADP-head becoming the same as other tubulins. Thus, the interaction potential between the kinesin head and MT along an MT protofilament in an ATPase cycle can be approximately shown in Fig. S1 (see Supplementary Information) and the mathematical description of the interaction potential is presented in Section S1 (see Supplementary Information).

(ii) When an MT-bound head is in ATP or ADP.Pi state there is a small free energy (denoted by *E*_NL_) to facilitate its neck linker (NL) docking into its motor domain^46^. The NL docking involves NL strand *β*9 and strand *β*0 of the motor domain forming a cover-neck bundle^47^ and only when *β*0 and *β*9 are in proximity can the cover-neck bundle formation take place. When an MT-bound kinesin head is in *ϕ* or ADP state, its NL is unable to dock^46^. In Section S2 (see Supplementary Information) we give mathematical description of the potential characterizing the effect of NL docking of the MT-bound head on the movement of the other tethered ADP-head.

(iii) An interaction is present between the two heads. When NL of the MT-bound head is undocked, a high binding energy (denoted by *E*_I1_) is present between the MT-bound head and detached ADP-head. When NL of the MT-bound head is docked, the binding energy (denoted by *E*_I2_) is reduced greatly, as argued before^48,49^ and supported by recent all-atom molecular dynamics simulations^50^. In Section S3 (see Supplementary Information) we give mathematical description of the potential of the interaction between the two heads. Note that with this argument and argument (ii), the biochemical data showing that upon the kinesin-1 dimer with both heads bound by ADP mixing with MT only half fraction of ADP molecules are released and addition of ATP molecules leads to release of other half fraction of ADP molecules^51^ can be explained well^49^.

As done before^33,34^, for simplicity of analysis, we do not consider the dissociation of the *ϕ*- or ATP- or ADP.Pi-head from MT under no or a small load, because in these states the kinesin head binds to MT strongly. Based on the model, the movement of tethered ADP-head relative to the other *ϕ*-, ATP- or ADP.Pi-head bound fixedly to MT can be described by Eqs. (S6) – (S11) (see Section S5 in Supplementary Information). When both ADP-heads are bound simultaneously to MT, the movement of one head relative to the other MT-bound head can still be described by Eqs. (S6) – (S11). When the two ADP-heads are bound together strongly, the movement of the MT-bound ADP-head relative to MT can be described by Eqs. (S12) – (S17) (see Section S6 in Supplementary Information).

Using Eqs. (S6) – (S17) we can simulate the mechanical motion of a kinesin head following Pi release relative to the other MT-bound kinesin head and the dissociation of the dimer from MT by using stochastic Runge–Kutta algorithm, as done before^33,34^. Then, we can simulate processive movement of the dimer by also considering continuous ATPase activities, which can be simulated using Monte-Carlo algorithm (see Section S7 in Supplementary Information).

As mentioned above, we consider that when one head is in *ϕ*, ATP or ADP.Pi state, the binding energy of the head to MT is so large that the dissociation of the head from the MT is negligibly small under no or a small load. This implies that only when both heads are in ADP state the dissociation of the motor is considered. In the calculations, when both heads move to positions that are away from the MT filament by more than 10 nm, the motor is considered to dissociate from MT^33,34^.

From a simulated trace of the displacement of the center of mass of the dimer versus time, the total displacement (or run length) of the trace can be obtained and the velocity of the trace can be calculated by dividing the total displacement by the total time before dissociation^33,34^. The mean run length and mean velocity are computed statistically using about 500 simulated traces.

### Intermolecular interaction between two neck coil segments and intramolecular interaction of residues near the N-terminus of neck coil segment with the head during cargo-mediated dimerization of kinesin-3

Before cargo binding, we make following argument for kinesin-3. The intramolecular interaction of residues near the N-terminus of neck coil (NC) segment (called N-residues) with the head inhibits its ATPase activity (concretely, ATP hydrolysis and Pi release), with the ATPase rate equal to 0 (Fig. 1a’). In other words, in the monomeric form kinesin-3 is inactive in the ATPase activity, which is consistent with the experimental data showing that kinesin-3 monomer, although can bind to MT, is inactive in motility^23^.

After cargo binding, we make following argument. Firstly, the C-terminal residue (adjacent to the cargo) in one NC helix forms an intermolecular interaction with the corresponding residue in another NC helix (Fig. 1b’ and c’). Then, the intermolecular interactions between residues in the two helixes form successively. Thus, the coiled-coil formation between two NC helixes is composed of multiple (*n* > 1) rate-limiting steps, with each rate-limiting step corresponding to the formation of the intermolecular interaction between a pair of residues in the two helixes. This implies that the cargo-mediated dimerization process, which begins with the formation of the intermolecular interaction between two C-terminal residues in two NC helixes and ends with the formation of the intermolecular interaction between N-residues in the two helixes, is composed of multiple rate-limiting steps. Moreover, we have the following expectation of the intramolecular interaction between the N-residues and head during the period after formation of the intermolecular interaction between two C-terminal residues in two NC helixes and before formation of the intermolecular interaction between N-residues in the two helixes. In the state with two heads bound to an MT filament, the stretching of the two NLs causes the N-residues to be away from their heads (Fig. 1b’). Thus, there is no intramolecular interaction between the N-residues and head, the two heads having the ATPase activity (Fig. 1b’). In the intermediate (INT) state, the N-residues are close to the head (Fig. 1c’). Thus, the intramolecular interaction between them is present, inhibiting the ATPase activity of the MT-bound head (Fig. 1c’). After formation of the stable coiled-coil between two NC helixes (Fig. 1d’ and e’), the formation of the intermolecular interaction between N-residues in the two helixes prevents the intramolecular interaction between the N-residues and head. Thus, the MT-bound head has the ATPase activity in both INT state (Fig. 1e’) and state with two heads bound to MT (Fig. 1d’).

### The choice of parameter values

In this work, we focus on two types of kinesin: kinesin-1 and kinesin-3. Their parameter values are chosen as follows.

#### Parameter values of kinesin-1

First, we consider kinesin-1. We take similar parameter values for kinesin-1 to those taken in our previous works^33,34^. We take *t*_*r*_ = 10 μs and *E*_w2_ = 39*k*_B_*T*. We take *E*_w1_ ≤ 18*k*_B_*T* (varying value of *E*_w1_ has little effect on our results provided *E*_w1_ ≤ 18*k*_B_*T*). Provided *E*_I1_ ≥ 40*k*_B_*T* and *E*_I2_ ≤ 20*k*_B_*T*, varying values of *E*_I1_ and *E*_I2_ has little effect on our results. We take NL-docking energy *E*_NL_ ≥ 4*k*_B_*T*, a small value that is consistent with the available experimental data^46^. In INT state, we take NL-docking rate *k*_NL_ = 800 s^−1^ when the MT-bound head is in ATP or ADP.Pi state and *k*_NL_ = 0 in *ϕ* or ADP state. This value of *k*_NL_ = 800 s^−1^ is consistent with the biochemical data^52^. We take the rate of ATP hydrolysis and Pi release of the trailing head with the forward NL orientation, ${k}_{{\rm{c}}}^{({\rm{trail}})}$ = *k*_c_ = 140 ${{\rm{s}}}^{-1}$, which is consistent with the biochemical data^53^. The rate of ATP hydrolysis and Pi release of the leading head without the forward NL orientation is taken to be ${k}_{{\rm{c}}}^{({\rm{lead}})}$ = ${k}_{{\rm{c}}}/\rho$, with $\rho$ = 40. In INT state, the rate of ATP hydrolysis and Pi release of the MT-bound head without the forward NL orientation is also ${k}_{{\rm{c}}}^{(\mathrm{int})}$ = ${k}_{{\rm{c}}}/\rho$. The fact that the head with the forward NL orientation has a much larger rate of ATP hydrolysis and Pi release than the head without the forward NL orientation can be understood as follows. With the forward orientation the NL can interact with the head and the interaction enhances the rate. This is consistent with the experimental data showing that by deleting NL (implying that the interaction of the NL with the head is removed) the ATPase rate is reduced greatly while the ADP-release rate is unaffected^54^. When ADP-head is bound to MT, the ADP-release rate *k*_D_* = *350 ${{\rm{s}}}^{-1}$, which is consistent with the biochemical data^53^. When ADP-head is detached from MT, without MT stimulation the ADP-release rate *k*_D_* = *0. We take the second-order rate constant of ATP binding, *k*_b_ = 2 ${\mu {\rm{M}}}^{-1}\cdot {{\rm{s}}}^{-1}$, which is consistent with the biochemical data^53^. The above parameter values for kinesin-1 are listed in Table 1 (noting that some parameters whose values are not required to have definite values are not included).

**Table 1:** Values of parameters for kinesin-1 and kinesin-3.

Parameter	Value	
	Kinesin-1	Kinesin-3
*t*_r_ (μs)	10	10
*E*_w1_	≤18*k*_B_*T*	≤18*k*_B_*T*
*E*_w2_	39*k*_B_*T*	≥45*k*_B_*T*
*k*_NL_ (s^−1^)	800	3400
*k*_c_ (s^−1^) (NL pointing forward)	140	270
*k*_*c*_^(lead)^ (s^−1^) (NL not pointing forward)	*k*_c_/40	*k*_c_/20
*k*_D_ (s^−1^)	350	350
*k*_b_ (μM^−1^ s^−1^)	2	2
*k*_cc_ (s^−1^)	—	0.17

Symbol “–” denotes that the value is not required.

As mentioned above, values of parameters *k*_NL_, *k*_c_, *k*_D_ and *k*_b_ are consistent with the available biochemical data^52,53^, while values of other parameters *t*_r_, *E*_w1_, *E*_w2_ and $\rho$ are taken as done in our previous works^33,34^, with which the available single-molecule data on the force dependence of mean velocity and run length (in particular the dramatic asymmetry of mean run length with respect to the force direction) for kinesin-1^27,35^ were reproduced quantitatively^33,34^.

#### Parameter values of kinesin-3

Then, we consider kinesin-3. Here, we focus mainly on mammalian kinesin-3 from subfamilies KIF1, KIF13, and KIF16, as used in the experiments by Verhey and his coworkers^23,31,32^. Since kinesin-3 head has a larger binding energy to MT than kiensin-1 head^31,32^, we take *E*_w2_ ≥ 45*k*_B_*T* for kinesin-3, which is larger than that for kinesin-1.

Denoting by *t* = 0 the moment when the intermolecular interaction forms between two C-terminal residues in the two NC helixes and *t* = *τ* the moment when the intermolecular interaction forms between N-residues in the two helixes, the distribution of time *τ* has the Gamma form^55^

1 $$f(\tau )=\frac{{k}_{0}^{n}{\tau }^{n-1}{e}^{-{k}_{0}\tau }}{(n-1)!},$$

where *n* is the number of rate-limiting steps of the coiled-coil formation between two NC helixes and *k*_0_ is the rate constant of each rate-limiting step. Note here that for simplicity of treatment but without loss of generality, we take each rate-limiting step having a constant rate. Before *τ*, in the state with two heads bound to MT the rates of ATP hydrolysis and Pi release of the trailing and leading heads are *k*_c_ and ${k}_{{\rm{c}}}/\rho$, respectively, and in INT state the rate of ATP hydrolysis and Pi release of the MT-bound head is 0. After *τ*, in the state with two heads bound to MT the rates of ATP hydrolysis and Pi release of the trailing and leading heads are *k*_c_ and ${k}_{{\rm{c}}}/\rho$, respectively, and in INT state the rate of ATP hydrolysis and Pi release of the MT-bound head is ${k}_{{\rm{c}}}/\rho$. In the calculation, we take *k*_c_ = 270 ${{\rm{s}}}^{-1}$ and $\rho$ = 20. The NL-docking rate is taken to be *k*_NL_ = 3400 ${{\rm{s}}}^{-1}$ when the MT-bound head is in ATP or ADP.Pi state and *k*_NL_ = 0 in $\varphi$ or ADP state. We take *n* as a variable parameter, with *k*_0_ = *nk*_cc_ and *k*_cc_* = *0.17 ${{\rm{s}}}^{-1}$, where 1/*k*_cc_ corresponds to the mean time of complete formation of the coiled-coil between the two NC helixes.

Except for the above parameters, we take all other parameters for kinesin-3 having the same values as those for kinesin-1. For clarity, the parameter values for kinesin-3 are also listed in Table 1.

## Results

### Velocity and run length distributions of dimeric kinesin-1

**Figure 2:**
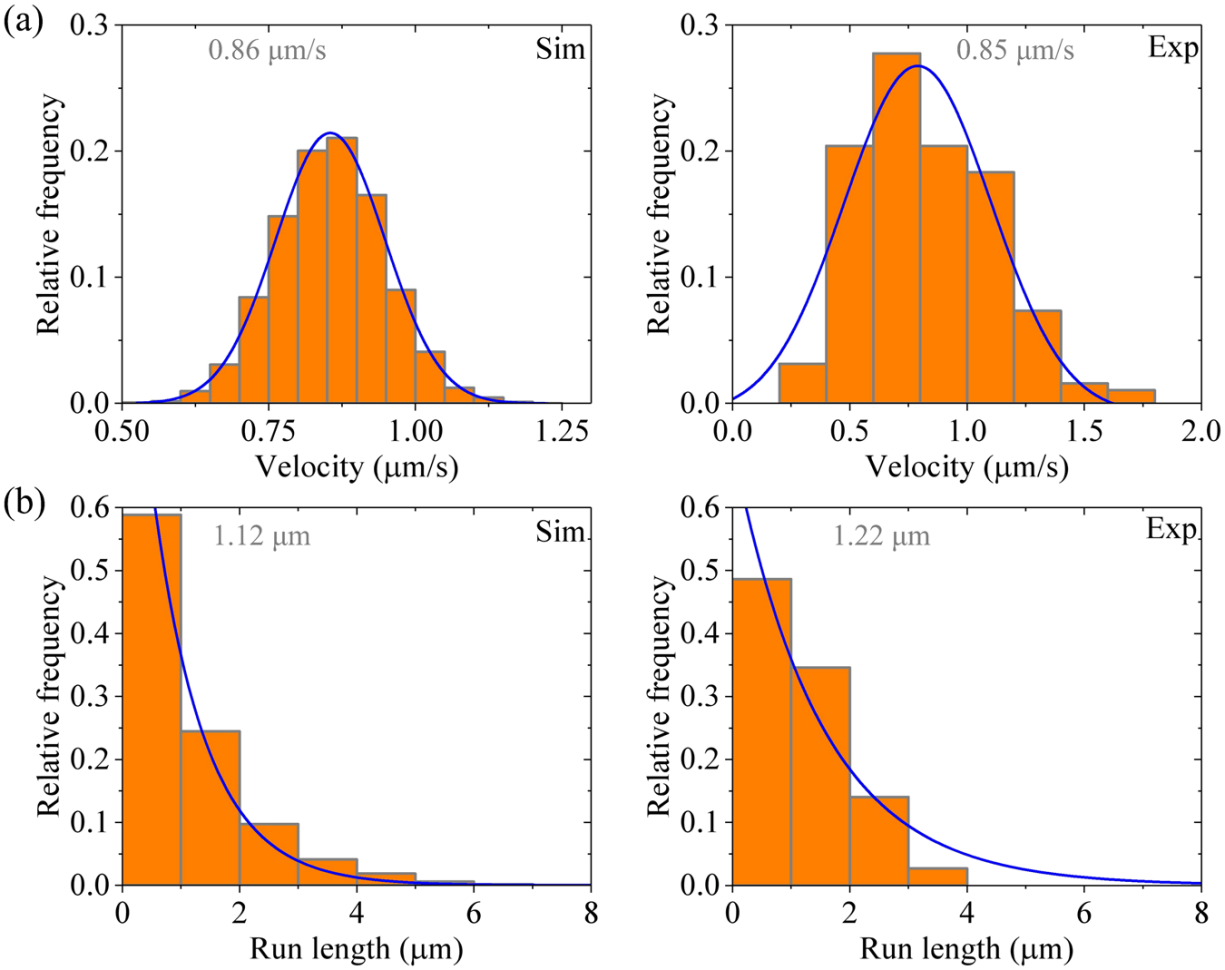
Results for dimeric kinesin-1 at saturating ATP. Left panels are data calculated with parameter values given in Table 1 for kinesin-1, while right panels are experimental data taken from Soppina *et al*.^23^. The mean values of the data are indicated. **(a)** Velocity distribution. Lines are Gaussian fits. **(b)** Run length distribution. Lines are single-exponential fits.

First, we consider kinesin-1 dimer at saturating ATP (2 mM). In Fig. 2a,b (left panels) we show the calculated results for distribution of velocities and that of run lengths, respectively. For comparisons, in Fig. 2 (right panels) we show the corresponding experimental data of Soppina *et al*.^23^. The calculated and experimental data are in quantitative agreement with each other. The data show that the velocity distribution has approximately a Gaussian form and the run length distribution has a single-exponential form. The Gaussian distribution of velocities is understandable easily, because under no load the backward stepping rate is negligibly small compared with the forward stepping rate^24^. The single-exponential distribution of run lengths can be understood as follows.

As noted from Fig. 1 and discussed before^33,34^, in a mechanochemical coupling cycle the dissociation of the motor from MT occurs mainly in two periods when the MT-bound head in INT state binds weakly to MT. One period (called Period I) is that when the MT-bound head in ADP state has an affinity *E*_w1_ for MT, namely, the period from the moment when Pi is released from the MT-bound head through the moment when the affinity of the ADP-head for the local MT tubulin is changed from *E*_w1_ to *E*_w2_ (Fig. 1f, state shaded in green). The other period (called Period II) is that when the MT-bound head in ADP state has an affinity *E*_w2_ for MT (Fig. 1g, state shaded in light red). The occurrence of Period I is determined by the rate of ATP hydrolysis and Pi release of the MT-bound head relative to that of its NL docking in INT state, while the occurrence of Period II is determined by the rate of ADP release from the leading head relative to the rate of ATP hydrolysis and Pi release of the trailing head. In Period I, due to the small affinity *E*_w1_ for MT, the head has a very large (~ 100%) probability to dissociate within time *t*_r_. In Period II, with the weak affinity *E*_w2_ for MT, the head also has a large (<100%) probability to dissociate before ADP release within time 1/*k*_D_. For the case of kinesin-1 with parameter values given in Table 1, both the dissociation occurring during Period I and that occurring during Period II have contributions to the overall dissociation, with the latter having a larger contribution than the former. Moreover, the dissociation probabilities in both periods have constant values in each step. Thus, the run length distribution is expected to have a single-exponential form.

### Velocity and run length distributions of dimerized kinesin-3

**Figure 3:**
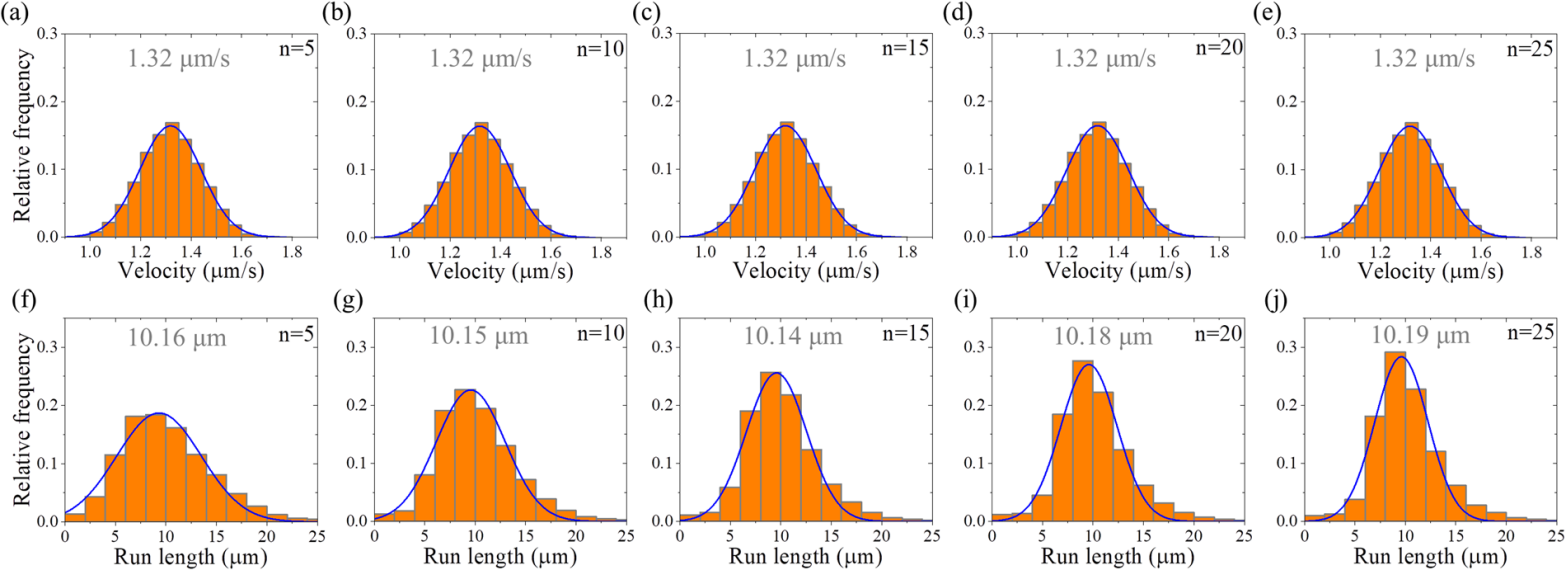
Results for dimerized kinesin-3 at saturating ATP. The data are calculated with parameter values given in Table 1 for kinesin-3 and different values of *n*. The mean values of the data are indicated. **(a**–**e)** Velocity distribution. Lines are Gaussian fits. **(f**–**j**) Run length distribution. Lines are Gaussian fits.

**Figure 4:**
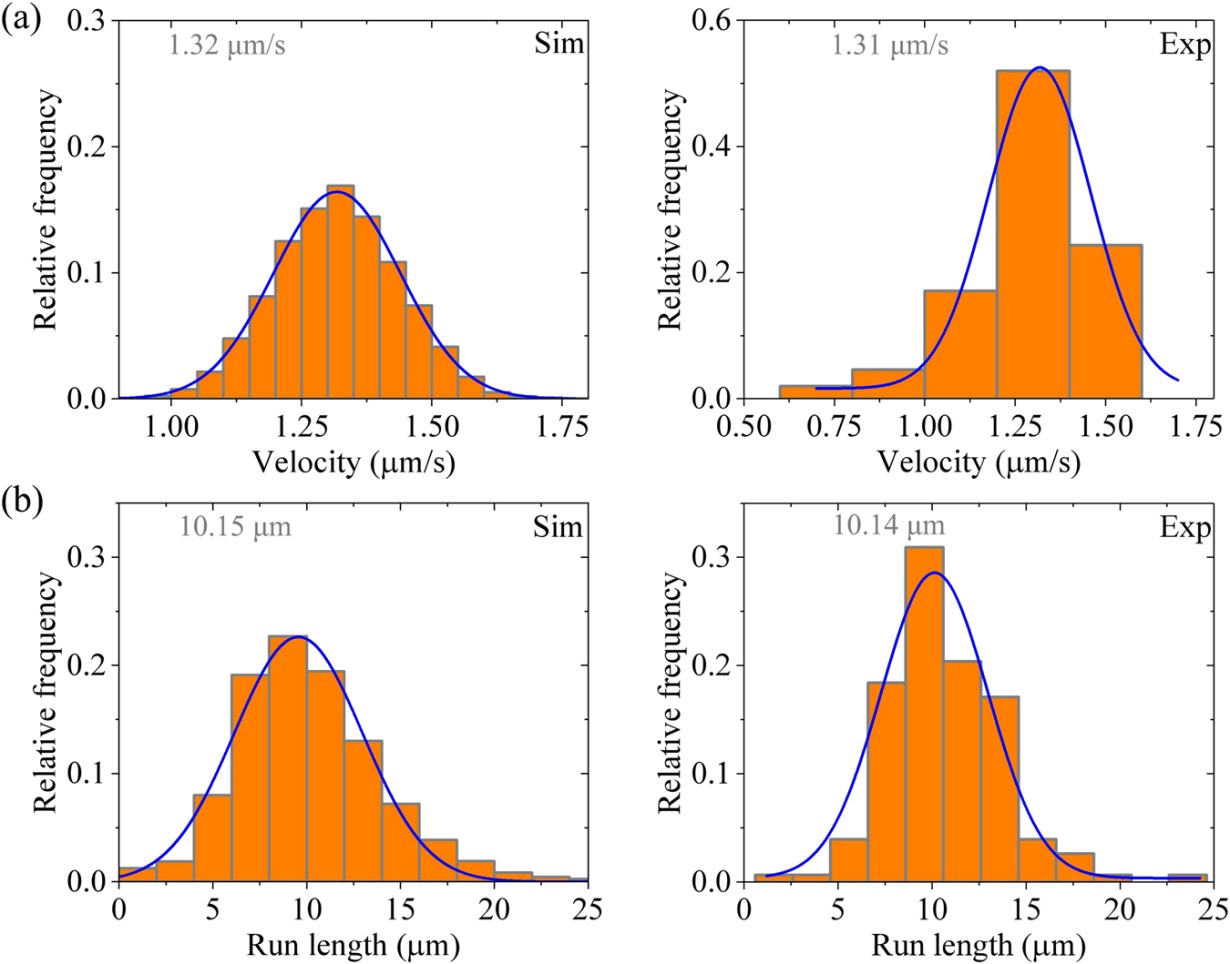
Results for dimerized kinesin-3 at saturating ATP. Left panels are data calculated with parameter values given in Table 1 for kinesin-3 and *n* = 10, while right panels are experimental data for KIF13B(1–412ΔP) taken from Soppina *et al*.^23^. The mean values of the data are indicated. **(a)** Velocity distribution. Lines are Gaussian fits. **(b)** Run length distribution. Lines are Gaussian fits.

Then, we consider kinesin-3 dimer at saturating ATP (2 mM). In Fig. 3 we show the calculated results of velocity distribution (Fig. 3a–e, upper panels) and run length distribution (Fig. 3f–j, lower panels) for different values of *n*. It is seen that as for the case of kinesin-1, the velocity distribution for kinesin-3 also has approximately a Gaussian form. Both the mean value of velocity and width of velocity distribution are independent of *n*. Interestingly, the run length distribution for kinesin-3 has approximately a Gaussian form rather than a single-exponential form. Although the mean value of run lengths is nearly independent of *n*, the width of run length distribution decreases with the increase of *n*. In particular, it is noted that when *n* = 10, the width of run length distribution is in good agreement with the experimental data^23^ (Fig. 4). This implies that there are about 10 rate-limiting steps for the coiled-coil formation between two NC helixes. Thus, in following calculations we fix *n* = 10.

The approximate Gaussian distribution of run lengths can be understood as follows. In our model, the value of *E*_w2_ determines only the probability of dissociation occurring during Period II. Our calculations show that the large value of *E*_w2_ ≥ 45*k*_B_*T* gives the probability of dissociation occurring during Period II to be negligibly small. Thus, the run length for dimerized kinesin-3 is determined only by the dissociation occurring during Period I. Before the rate of ATP hydrolysis and Pi release of the MT-bound head in INT state changes from 0 to ${k}_{{\rm{c}}}/\rho$, Period I cannot occur and thus nearly no dissociation can occur. After the rate of ATP hydrolysis and Pi release of the MT-bound head changes to ${k}_{{\rm{c}}}/\rho$ at *t* = *τ*, Period I can occur. Once Period I occurring, the MT-bound head, which is bound strongly with the detached ADP-head, has nearly 100% probability to dissociate from the potential well of depth *E*_w1_ (≤ 18*k*_B_*T*) within time *t*_r_. Thus, from the Gamma distribution of *τ* [Eq. (1)] an approximate Gaussian distribution of run lengths would be expected at large *n*. However, it is noted that since $\rho$ = 20 is much larger than 1, implying that the rate of ATP hydrolysis and Pi release of the MT-bound head in INT state is much smaller than that of the trailing head, the change of the rate of ATP hydrolysis and Pi release of the MT-bound head in INT state from 0 to ${k}_{{\rm{c}}}/\rho$ has nearly no effect on velocity.

As mentioned above, our calculations show that provided that *E*_w2_ ≥45*k*_B_*T*, changing *E*_w2_ has little effect on our results of velocity and run length. This provides an explanation of the experimental data showing that although the K-loop enhances the binding affinity to MT, the mutation of the K-loop has little effect on the velocity and run length of kinesin-3 dimers^32^. This is understandable easily. For *E*_w2_ ≥45*k*_B_*T*, the dissociation during Period II is negligibly small and thus changing value of *E*_w2_ caused by the mutation of the K-loop has nearly no effect on the overall dissociation. On the other hand, provided that *E*_w1_ ≤18*k*_B_*T*, once Period I occurring the MT-bound head has nearly 100% probability to dissociate from the potential well of depth *E*_w1_ within time *t*_r_. Thus, the change of *E*_w1_ caused by the mutation of the K-loop also has no effect on the dissociation.

**Figure 5:**
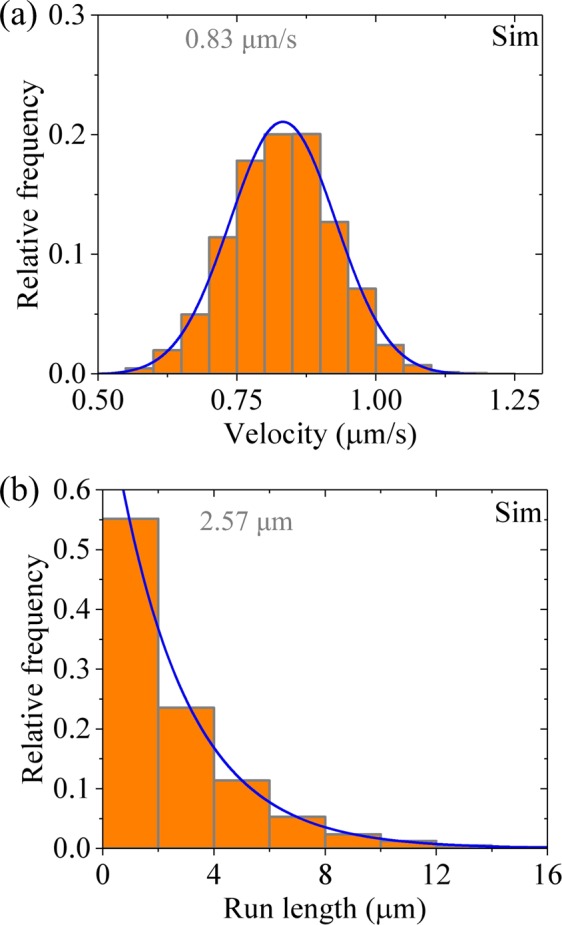
Results for dimeric kinesin-1 at saturating ATP. The data are calculated with parameter values given in Table 1 for kinesin-1 except for *E*_w2_ ≥ 45*k*_B_*T*. The mean values of the data are indicated. (**a)** Velocity distribution. Line is the Gaussian fit. **(b)** Run length distribution. Line is the single-exponential fit.

To examine whether compared to kinesin-1 the enhancement of *E*_w2_ is sufficient to give the superprocessivity of kinesin-3, we calculate the distribution of velocities and that of run lengths with parameter values given in Table 1 for kinesin-1 except with *E*_w2_ ≥45*k*_B_*T* (Fig. 5). Comparing Fig. 5b with Fig. 2b and with Fig. 4b, it is seen that although the enhancement of *E*_w2_ increases the mean run length by about 2.3-fold the mean run length is still about 4-fold smaller than that of kinesin-3. In addition, the run length distribution (Fig. 5b) still has a single-exponential form. This is because with *E*_w2_ ≥45*k*_B_*T* although the dissociation occurring during Period II is negligibly small, the large dissociation probability during Period I, which can now occur at *t* = 0, prevents the dimers from becoming the supperprocessive motors. Thus, we conclude that compared to kinesin-1 the enhancement of *E*_w2_ is insufficient to give the superprocessive motion of kinesin-3. The superprocessivity of kinesin-3 is due to both the slow transition of the rate of ATP hydrolysis and Pi release of the MT-bound head in INT state from 0 to ${k}_{{\rm{c}}}/\rho$ and the large value of *E*_w2_.

**Figure 6:**
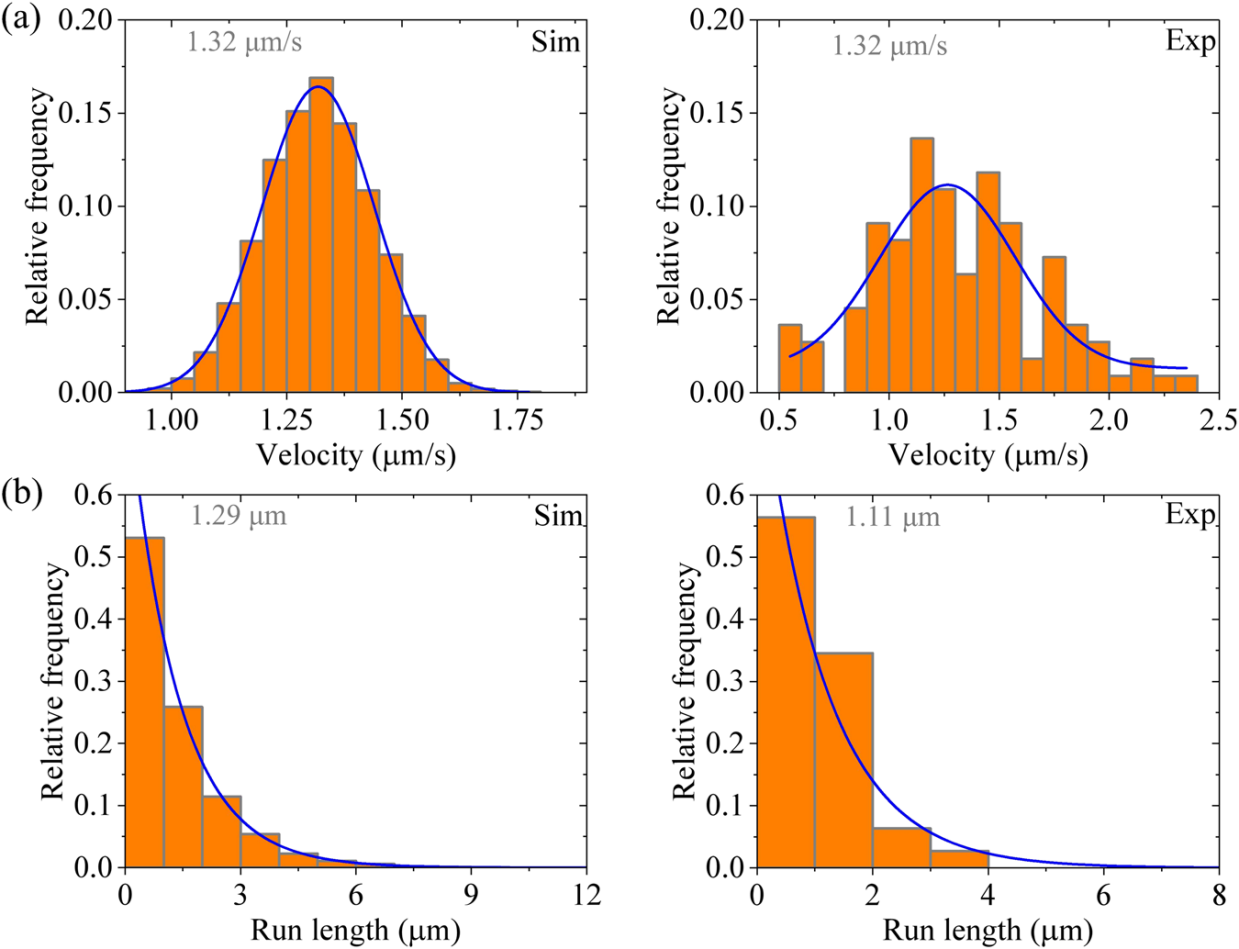
Results for for dimerized kinesin-3 at saturating ATP. Left panels are data calculated with parameter values given in Table 1 for kinesin-3 and *n* = 10 except for *E*_w2_ = 39*k*_B_*T*, while right panels are experimental data for L8 mutant of KIF13B (ΔP391) taken from Scarabelli *et al*.^31^. The mean values of the data are indicated. **(a)** Velocity distribution. Lines are Gaussian fits. **(b)** Run length distribution. Lines are single-exponential fits.

To explain the experimental data on the effect of reducing *E*_w2_ on run length of kinesin-3 by mutating residues that have large effects on binding affinity to MT^31^, we calculate the distribution of run lengths with *E*_w2_ = 39*k*_B_*T* while with other parameter values being given in Table 1 for kinesin-3. The calculated results are shown in Fig. 6 (left panels). For comparisons, the experimental data for L8 mutant of KIF13B (ΔP391) in Scarabelli *et al*.^31^ are also shown in Fig. 6 (right panels). It is seen that the calculated data reproduce well the experimental data. That the run length distribution has a single-exponential form can be understood as follows. The value of *E*_w2_ = 39*k*_B_*T* gives a large dissociation probability occurring during Period II, giving the dimer to dissociate from MT before the rate of ATP hydrolysis and Pi release of the MT-bound head in INT state changes from 0 to ${k}_{{\rm{c}}}/\rho$. On the other hand, with the rate of ATP hydrolysis and Pi release equal to zero, no dissociation can occur during Period I and thus the dissociation arises solely from that occurring during Period II. Since in each step the probability of dissociation occurring during Period II is constant, it is expected that the run length distribution has a single-exponential form.

**Figure 7:**
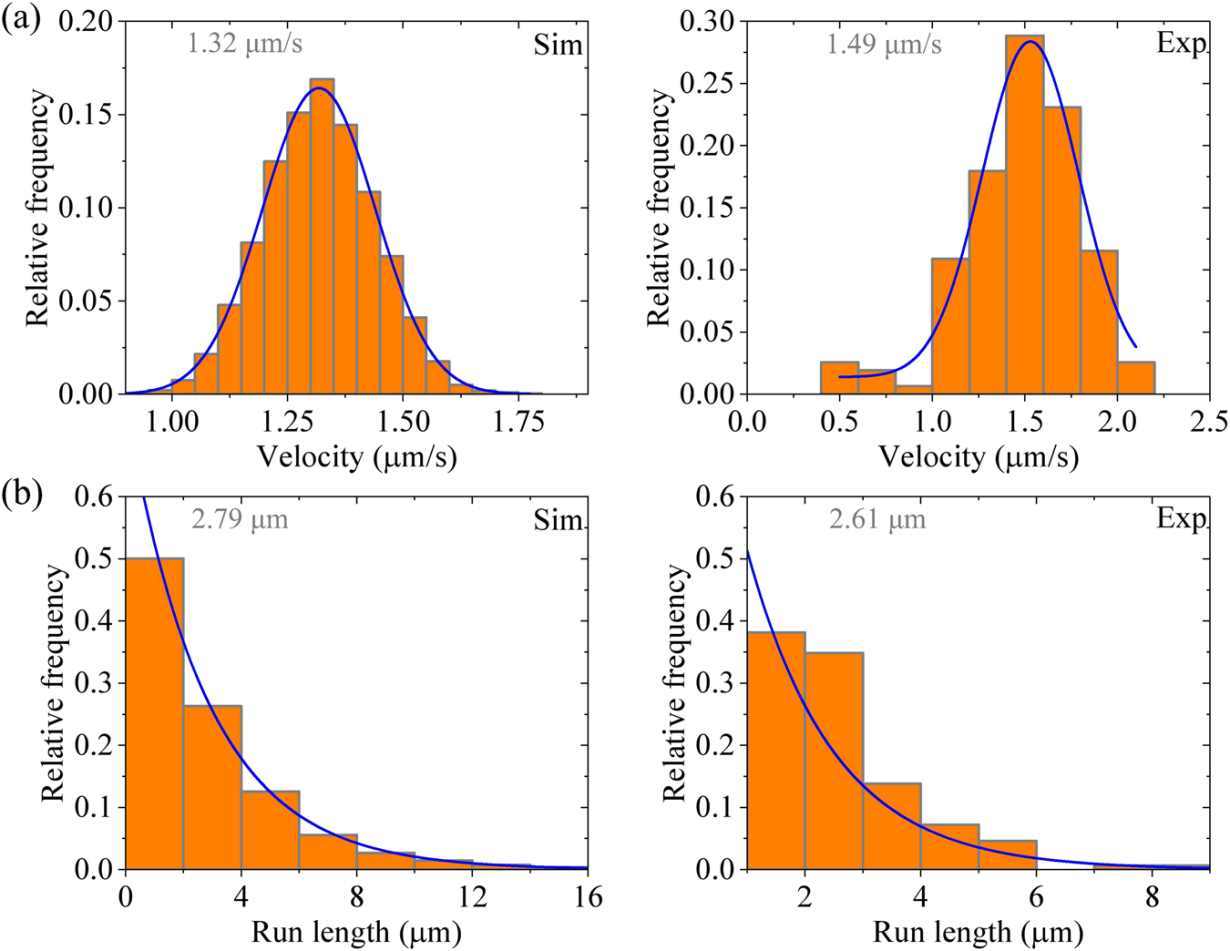
Results for dimerized KIF1A(1–393) at saturating ATP. It is considered that at *t* = 0 the rate of ATP hydrolysis and Pi release of the MT-bound head in INT state has the non-zero value of ${k}_{{\rm{c}}}/\rho$. Left panels are data calculated with parameter values given in Table 1 for kinesin-3 and *n* = 10, while right panels are experimental data for KIF1A(1–393) taken from Soppina *et al*.^23^. The mean values of the data are indicated. **(a)** Velocity distribution. Lines are Gaussian fits. **(b)** Run length distribution. Lines are single-exponential fits.

Now, we give an explanation of the experimental data on KIF1A(1–393) dimer showing only moderately processive with an average run length of ∼2.5 μm and a single-exponential distribution of run lengths. In KIF1A(1–393), since the NC segment could be unstable, no interaction between the NC and head would be present. Thus, we consider that when the cargo-mediated dimerization begins at *t* = 0, the rate of ATP hydrolysis and Pi release of the MT-bound head in INT state has the non-zero value of ${k}_{{\rm{c}}}/\rho$. With this consideration and parameter values for kinesin-3 given in Table 1, we calculate the velocity and run length distributions, with the results being shown in Fig. 7 (left panels). For comparison, the corresponding experimental data for KIF1A(1–393)^23^ are shown in Fig. 7 (right panels). It is seen that the calculated data are in good agreement with the experimental data.

### Velocity and run length of dimeric kinesin-1 and kinesin-3 at low ATP concentrations

In the above, we made studies at saturating ATP, explaining quantitatively the available experimental data. In this section, we make studies at non-saturating ATP, providing some predicted results.

**Figure 8:**
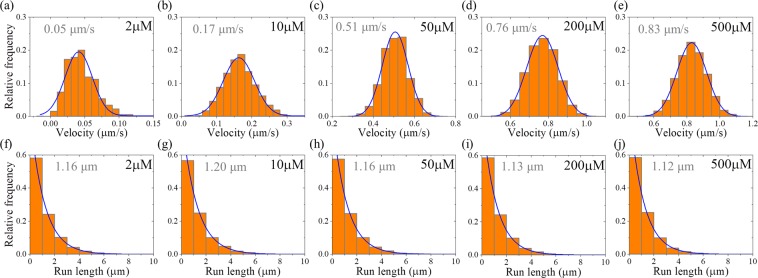
Results for dimeric kinesin-1 at non-saturating ATP concentrations. The data are calculated with parameter values given in Table 1 for kinesin-1. The mean values of the data are indicated. **(a**–**e)** Velocity distribution. Lines are Gaussian fits. **(f**–**j)** Run length distribution. Lines are single-exponential fits.

First, we consider kinesin-1 dimer. In Fig. 8 we show the calculated results of velocity distribution (Fig. 8a–e, upper panels) and run length distribution (Fig. 8f–j, lower panels) at different ATP concentrations. It is seen that at any ATP concentration, the velocity distribution has the Gaussian form and the run length distribution has the single-exponential form. The mean velocity increases with the increase of ATP concentration, and the mean run length is independent of ATP concentration, which is consistent with the available single-molecule experimental data^27^.

**Figure 9:**
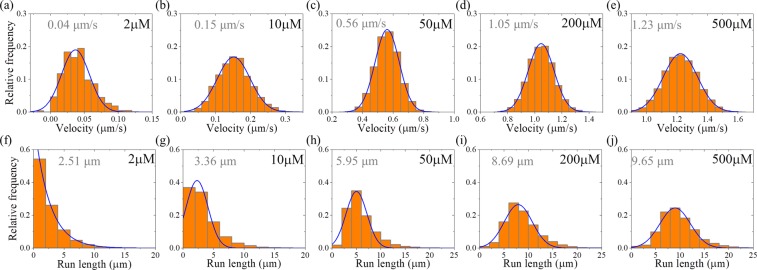
Results for dimerized kinesin-3 at non-saturating ATP concentrations. The data are calculated with parameter values given in Table 1 for kinesin-3 and *n* = 10. The mean values of the data are indicated. **(a**–**e)** Velocity distribution. Lines are Gaussian fits. **(f**–**j)** Run length distribution. Line in (f) for [ATP] = 2 μM is the single-exponential fit, while lines in (**g**–**j**) for other values of [ATP] are the Gaussian fits.

Second, we consider kinesin-3 dimer. In Fig. 9 we show the calculated results of velocity distribution (Fig. 9a–e, upper panels) and run length distribution (Fig. 9f–j, lower panels) at different ATP concentrations. It is seen that at any ATP concentration, the velocity distribution has the Gaussian form. However, as ATP concentration decreases, the run length distribution deviates from the Gaussian form. Moreover, both the mean velocity and mean run length decrease with the decrease of ATP concentration.

### Force dependence of run length for dimerized kinesin-3

Up to now, we have made studies under no load. The dependencies of the mean velocity and run length on load for kinesin-1 have been calculated before^33,34^. Thus, in this section we make only studies for dimerized kinesin-3 under a backward load, *F*, on the cargo, providing some predicted results and giving an explanation of the available single-molecule data for another species of dimerized kinesin-3, U356-Kstalk-GFP, which was created by joining the kinesin-1 neck coiled-coil and stalk to Unc104 motor domain^22^. In this section, we consider saturating ATP and *F* in the range smaller than the stall force so that the dissociation can be neglected when at least one head binds strongly to MT^56^.

Under the backward load on the cargo, we make following considerations. (i) The load on the NC helixes would disrupt the intramolecular interaction between the N-residues and kinesin-3 head in INT state before the formation of the intermolecular interaction between N-residues in the two helixes. This implies that at beginning of the processive movement of the dimerized kinesin-3, i.e., at *t* = 0, the ATPase activity of the MT-bound head in INT state is not inhibited, with the rate of ATP hydrolysis and Pi release being ${k}_{{\rm{c}}}/\rho$ all the time. (ii) In INT state, when the NL of the MT-bound head is stretched to a length *l*_NL_ > 2.8 nm under the backward load, the rate constant of NL docking ${k}_{{\rm{N}}{\rm{L}}}^{({\rm{B}})}={k}_{{\rm{N}}{\rm{L}}}/\lambda$ (*λ* ≥ 1), as done before^34^. Here, we take two values of *λ*, with *λ* = 2 and 5, for the calculations. The equations for the calculations under the load are the same as those described previously^33,34^.

**Figure 10:**
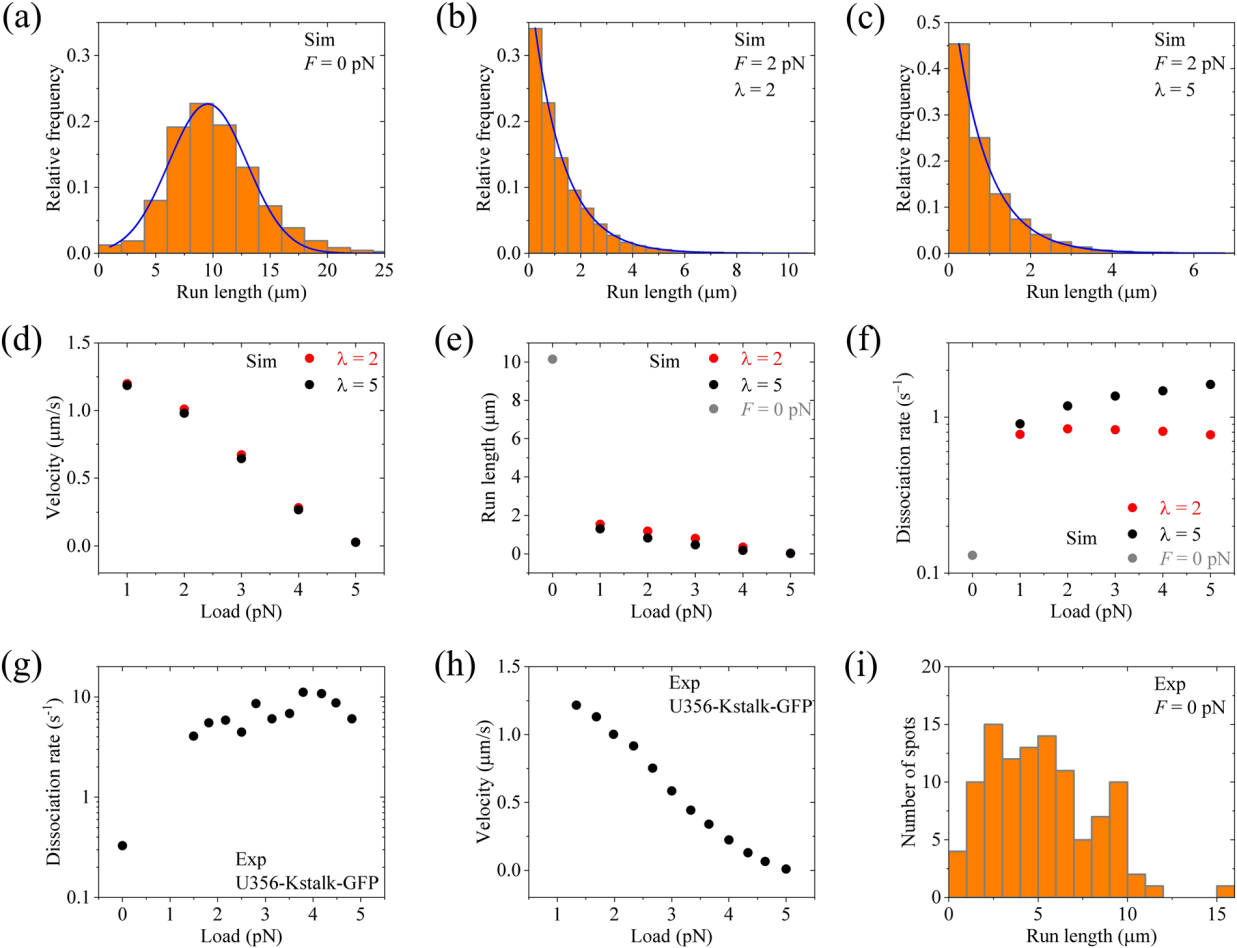
Results of the force dependencies of velocity and run length for dimerized kinesin-3 at saturating ATP. The theoretical data are calculated with *E*_NL_ = 5*k*_B_*T* and other parameter values given in Table 1 for kinesin-3 and *n* = 10. The experimental data are taken from Tomishige *et al*.^22^. **(a)** Calculated run length distribution under no load. **(b)** Calculated run length distribution under *F* = 2 pN for *λ* = 2. **(c)** Calculated run length distribution under *F* = 2 pN for *λ* = 5. **(d)** Calculated mean velocity versus load. **(e)** Calculated mean run length versus load. **(f)** Calculated dissociation rate versus load. **(g)** Experimentally measured dissociation rate versus load for U356-Kstalk-GFP. **(h)** Experimentally measured velocity versus load for U356-Kstalk-GFP. **(i)** Experimentally measured run length distribution under no load for U356-Kstalk-GFP.

With the above two considerations, taking *E*_NL_ = 5*k*_B_*T* and other parameters as given in Table 1 for kinesin-3, we calculate the run length and velocity for different values of the backward load *F*. Note here that the rate constants of ATPase activity are independent of *F*, as done before^48,49,57,58^. In Fig. 10a we show the calculated results of the run length distribution under no load (same as the left panel of Fig. 4b). In Fig. 10b and c we show the calculated results of the run length distribution under *F* = 2 pN for *λ* = 2 and 5, respectively. It is seen that while under no load the distribution of run lengths has evidently a non-single-exponential form (Fig. 10a), under the load the distribution becomes single exponential (Fig. 10b,c). In Fig. 10d and e we show the calculated results of the mean velocity and mean run length versus load *F*. From Fig. 10e we see interestingly that even under a small backward load the run length decreases significantly relative to that under no load, and as the backward load increases the run length decreases slowly. This feature for kinesin-3 is in sharp contrast to that for kinesin-1, whose run length under a small backward load is reduced only slightly relative to that under no load^27,33–35^. In Fig. 10f we show the force dependence of dissociation rate *k*_d_ for kinesin-3, which is calculated from the mean detachment time (noting that *k*_d_ can also be obtained by dividing mean velocity by mean run length). From Fig. 10f it is seen that a small load can enhance the dissociation rate significantly, especially with the large value of *λ*. As the load increases, the dissociation rate changes only slightly. This feature is consistent with the available single-molecule data for U356-Kstalk-GFP (Fig. 10g)^22^. Moreover, it is noted that the calculated curve of velocity versus *F* (Fig. 10d) also resembles well the available experimental curve for U356-Kstalk-GFP (Fig. 10h)^22^. The measured run length distribution under no load for U356-Kstalk-GFP (Fig. 10i)^22^ also evidently has a non-single-exponential form, similar to the calculated one (Fig. 10a).

Based on our model, the great increase of the dissociation rate under a backward load relative to that under no load arises from two effects. One effect is that the load disrupts the intramolecular interaction between the N-residues and head in INT state before the formation of the intermolecular interaction between N-residues in the two helixes. This brings forward the time when Period I occurs, increasing the dissociation rate by *μ*-fold (*μ* > 1), as noted by comparing Fig. 4b (left panel) with Fig. 7b (left panel). The other effect is that under the backward load the rate constant of NL docking in the MT-bound head in INT state is reduced by *λ*-fold, increasing the occurrence probability of Period I by approximately *λ*-fold. Both effects are involved only in the dissociations during Period I. Since the dissociation during Period II is negligible, the two effects thus result in the total dissociation rate to increase by approximately *μλ*-fold.

**Figure 11:**
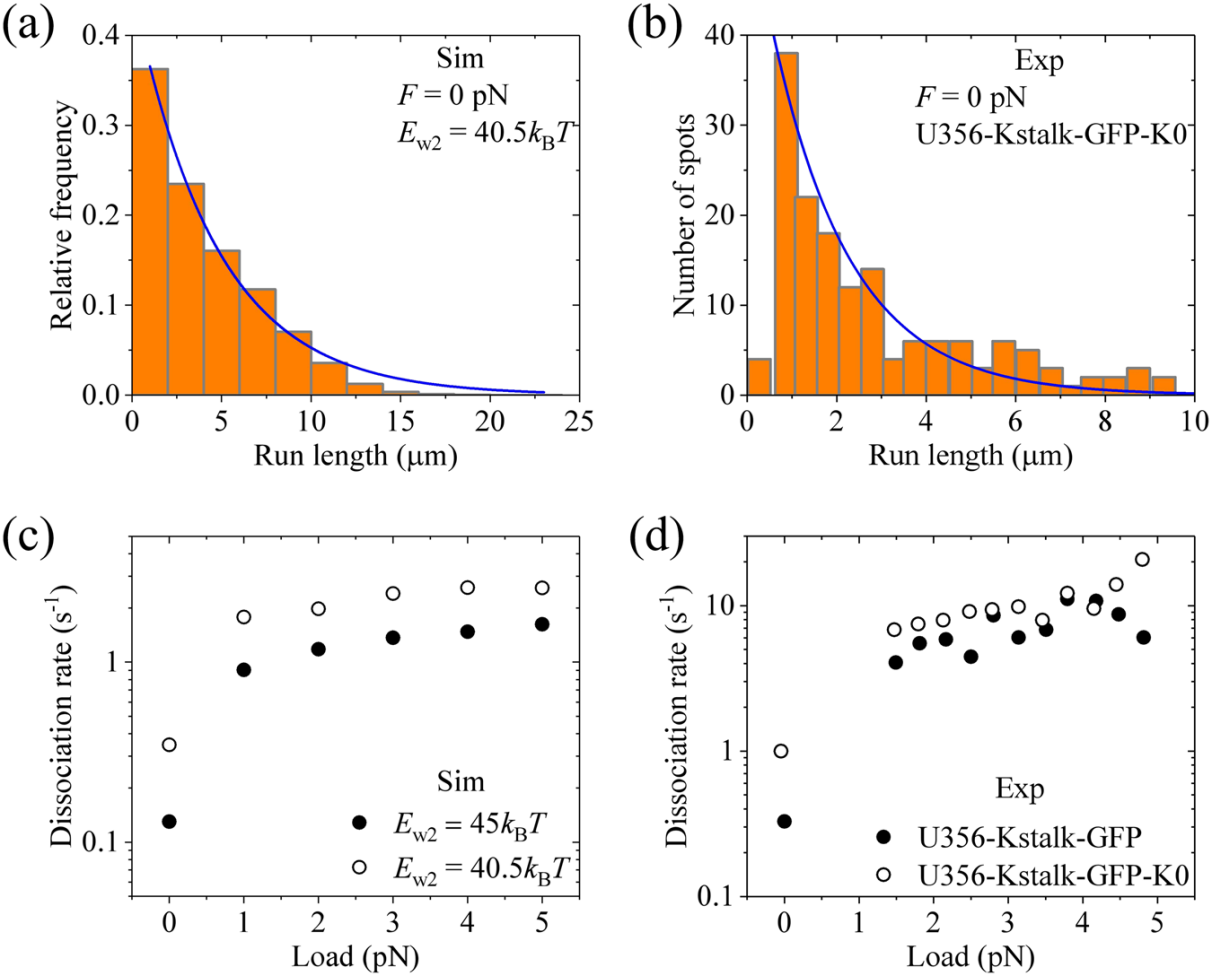
Results of the force dependencies of velocity and run length for dimerized kinesin-3 with reduced affinity *E*_w2_ at saturating ATP. The theoretical data are calculated with *E*_NL_ = 5*k*_B_*T* and other parameter values given in Table 1 for kinesin-3 and *n* = 10 except for *E*_w2_ = 40.5*k*_B_*T*. The experimental data are taken from Tomishige *et al*.^22^. **(a)** Calculated run length distribution under no load. **(b)** Experimentally measured run length distribution under no load for U356-Kstalk-GFP-K0. **(c)** Calculated dissociation rate versus load (unfilled circles). *λ* = 5. **(d)** Experimentally measured dissociation rate versus load for U356-Kstalk-GFP-K0 (unfilled circles). The filled circles in (**c**) are calculated data with *E*_w2_ ≥ 45*k*_B_*T* and *λ* = 5. The filled circles in (**d**) are experimental data for U356-Kstalk-GFP.

In addition, we consider that the dissociation during Period II can also occur. We take *E*_w2_ = 40.5*k*_B_*T*, which is smaller than *E*_w2_ ≥45*k*_B_*T*, while with other parameter values being given as just above in Fig. 10 for kinesin-3. Under no load, the distribution of run lengths with *E*_w2_ = 40.5*k*_B_*T* becomes single exponential and the mean run length is reduced greatly (Fig. 11a). This is consistent with the available experimental data for U356-Kstalk-GFP-K0 (Fig. 11b), where the K-loop in U356-Kstalk-GFP was swapped with that from kinesin-1^22^, implying that *E*_w2_ for U356-Kstalk-GFP-K0 is reduced relative to that for U356-Kstalk-GFP. The calculated results of dissociation rate versus *F* with *E*_w2_ = 40.5*k*_B_*T* are shown in Fig. 11c (unfilled circles, *λ* = 5), where for comparison the corresponding calculated data with *E*_w2_ ≥45*k*_B_*T* are re-shown (filled circles, *λ* = 5). From Fig. 11c, it is seen that while under no load the dissociation rate with *E*_w2_ = 40.5*k*_B_*T* is much larger than that with *E*_w2_ ≥45*k*_B_*T*, under loads the dissociation rates with *E*_w2_ = 40.5*k*_B_*T* become closer to those with *E*_w2_ ≥45*k*_B_*T*. These features are also consistent with the available single-molecule data for U356-Kstalk-GFP-K0 (Fig. 11d, unfilled circles) relative to those for U356-Kstalk-GFP (Fig. 11d, filled circles)^22^.

## Discussion

Soppina *et al*^23^. showed that in the monomeric state the full-length kinesin-3 is inactive in motility and only after the dimerization can the kinesin-3 move processively. By contrast, Okada *et al*^59,60^. and Oriola *et al*^61,62^. showed clearly that the truncated monomeric kinesin-3 KIF1A can move processively. Based on our proposed model, these contradictory results can be explained easily. In our model, in the monomeric state the formation of the intramolecular interaction between the N-residues in NC segment and the head inhibits the ATPase activity of the head, and the dimerization prevents the formation of the intramolecular interaction between the N-residues and the head, without inhibiting the ATPase activity of the head. In the monomeric state of the full-length kinesin-3 used by Soppina *et al*.^23^, the intramolecular interaction can form between the N-residues of NC segment and the head, inhibiting the ATPase activity of the head. By contrast, in the monomeric state of truncated kinesin-3 KIF1A (amino acids 1–382) used by Okada *et al*^59,60^. and Oriola *et al*.^61,62^, where the N-residues in NC segment are supposed to be deleted, the ATPase activity of the head is not inhibited. As a result, the single KIF1A monomer can move processively with the Brownian ratchet mechanism proposed before^43,59–63^.

As previous theoretical studies showed^24,25^, if a molecular motor has a constant rate to dissociate from its track during its processive movement or the motor has a constant probability to dissociate in each mechanochemical coupling cycle, a single-exponential distribution of run lengths is obtained. For kinesin-1 it is true that the motor has a constant dissociation rate, but for kinesin-3 this is not the case, as described as follows. As mentioned above, the dissociation of the kinesin dimer from MT can occur in two periods—Period I and Period II (see Fig. 1), and the motor has a constant probability to dissociate in each period. Thus, the dissociation rate of the kinesin dimer can be calculated by $\varepsilon ={k}_{{\rm{I}}}{P}_{{\rm{I}}}+{k}_{{\rm{II}}}{P}_{{\rm{II}}}$, where *k*_I_ is the occurrence rate of Period I, *P*_I_ the dissociation probability in Period I, *k*_II_ the occurrence rate of Period II and *P*_II_ the dissociation probability in Period II. Since *P*_I_ and *P*_II_ have constant values, it is thus seen that the non-exponential distribution of run lengths, which arises from a non-constant *ε*, requires *k*_I_ and/or *k*_II_ having non-constant values. For kinesin-1, both Period I and Period II occur with constant rates *k*_I_ and *k*_II_, respectively, giving a constant *ε* and thus a single-exponential distribution of run lengths. By contrast, for kinesin-3, Period I occurs with a non-constant rate *k*_I_, which is dictated by the non-exponential distribution of time $\tau$ when the intermolecular interaction between the N-residues in the two helixes is formed [see, Eq. (1)]. This gives a non-constant *ε* and thus a non-exponential distribution of run lengths. Note that for kinesin-3, since *P*_II_ $\approx$ 0, *k*_II_ has no effect on *ε*. Thus, it is only required that *k*_I_ has a non-constant value to give a non-constant *ε* and a non-exponential distribution of run lengths.

In summary, we provided quantitative explanations of the experimental data on dynamics of dimerized kinesin-3. The peculiar features of approximate Gaussian distribution of run lengths and superprocessivity for kinesin-3 are due to the reason that after cargo-mediated dimerization the ATPase activity of the MT-bound head in INT state is still inhibited for a time period after the heads become active in the two-heads-bound state. By contrast, for kinesin dimers such as kinesin-1, kinesin-2, kinesin-4, kinesin-7, etc., which have intrinsically stable coiled-coil structures and the ATPase activities of the MT-bound head in INT state are not inhibited, their run length distributions have the normally single-exponential form. Moreover, the force dependence of run length or dissociation rate for kinesin-3 was also studied, explaining the available experimental data showing contrast features of the force dependence of dissociation rate between kinesin-3 and kinesin-1.

## Supplementary information

Supplementary
