# Supplementary material for: Run length distribution of dimerized kinesin-3 molecular motors: comparison with dimeric kinesin-1
Source: Sci Rep. 2019 Nov 18;9:16973. doi: 10.1038/s41598-019-53550-2 (PMC6861319; doi:10.1038/s41598-019-53550-2)
Supplement: Supplementary file 1 — Supplementary [file 41598_2019_53550_MOESM1_ESM.pdf]

Supplementary Information

Run length distribution of dimerized kinesin-3 molecular motors: comparison with dimeric kinesin-1

Si-Kao Guo, Xiao-Xuan Shi, Peng-Ye Wang, Ping Xie *

Key Laboratory of Soft Matter Physics, Institute of Physics, Chinese Academy of Science, Beijing 100190, China

*Corresponding author:

E-mail address: pxie@aphy.iphy.ac.cn

SI text

S1. Potential of interaction between one kinesin head and MT in a mechanochemical coupling cycle

Based on argument (i) (see main text), we take the interaction potential of one kinesin head with MT as described as follows. In nucleotide-free state the kinesin head binds strongly to MT, with the interaction potential being written as $V_S(x,y,z,\alpha,\theta,\phi)=V_{Sx}(x)V_y(y)V_z(z)V_\alpha(\alpha)V_\theta(\theta)V_\phi(\phi)$, where coordinate *oxyz* is defined in Fig. S1a, α, θ and ϕ are angles characterizing respectively the nutation, rotation and precession motions (when the kinesin head is in the MT-binding site, α, θ and ϕ correspond to the angles of rotations in *xoz*, *xoy* and *yoz* planes, respectively). Term $V_{Sx}(x) < 0$ (with the maxima equal to zero) represents the interaction potential between the kinesin head and MT along a MT protofilament and is approximately shown in Fig. S1a. The period of $V_{Sx}(x)$, d = 8.2 nm, is equal to the distance between two successive binding sites on MT filaments. Here we take $V_{Sx}(x)$ in one periodicity having an asymmetric form here, with asymmetric ratio d_1/d_2 = 3/5, as done before [S1]. It should be mentioned that taking other forms for $V_{Sx}(x)$ (including the symmetric form) has little effect on the results for the dimer. Terms $V_y(y)=\exp\left[-(y-y_0)/A_y\right]$ and $V_z(z)=\exp\left(-|z-z_0|/A_z\right)$ denote the potential changes in the vertical and horizontal directions, respectively, with A_y and A_z characterizing the

interaction distances. Note that, due to the steric restriction of MT, the position of the kinesin head is confined to the region $y \geq y_0$. Terms $V_\alpha(\alpha)=\exp\left(-|\alpha-\alpha_0|/A_\alpha\right)$, $V_\theta(\theta)=\exp\left(-|\theta-\theta_0|/A_\theta\right)$ and $V_\phi(\phi)=\exp\left(-|\phi-\phi_0|/A_\phi\right)$ denote the potential changes arising from the head rotations, with A_α, A_θ and A_ϕ characterizing the interaction distances. Here, we define $y_0 = z_0 = \alpha_0 = \theta_0 = \phi_0 = 0$ in the state of the head bound to MT. These potential changes $V_y(y)$, $V_z(z)$, $V_\alpha(\alpha)$, $V_\theta(\theta)$ and $V_\phi(\phi)$ are similar to the Morse potential form that describes the van der Waals interaction. To be consistent with the Debye length that is in the order of 1 nm in solution, we take $A_y = A_z = 1$ nm and $r_{\text{head}}A_\alpha$ = $r_{\text{head}}A_\theta$ = $r_{\text{head}}A_\phi$ = 1 nm, where the kinesin head is approximately a sphere of radius $r_{\text{head}} = 2.5$ nm. After ATP binding and then hydrolysis to ADP.Pi the kinesin head remains bound strongly to MT, with the interaction potential still being approximately described by $V_{\text{S}}(x,y,z,\alpha,\theta,\phi)$.

Immediately after Pi release, the interaction potential becomes one that can be written as $V_{\text{W}}(x,y,z,\alpha,\theta)=V_{\text{W}x1}(x)V_y(y)V_z(z)V_\alpha(\alpha)V_\theta(\theta)V_\phi(\phi)$, with $V_{\text{W}x1}(x) < 0$ being approximately shown in Fig. S1b and $V_y(y)$, $V_z(z)$, $V_\alpha(\alpha)$, $V_\theta(\theta)$ and $V_\phi(\phi)$ being the same as those defined above. Note that immediately after Pi release the binding affinity (E_{w1}) of the kinesin head for the local binding on MT site, where the kinesin head in ADP.Pi state has just bound, is weaker than that (E_{w2}) at other binding sites. After a period of time t_r, the affinity of the local binding site on MT for ADP-head relaxes to the normal value and the interaction potential becomes $V_{\text{W}}(x,y,z,\alpha,\theta)=V_{\text{W}x2}(x)V_y(y)V_z(z)V_\alpha(\alpha)V_\theta(\theta)V_\phi(\phi)$, with $V_{\text{W}x2}(x)$ being shown approximately in Fig. S1c. Note that the weak binding affinity (E_{w2}) of ADP-kinesin for MT is smaller than the strong binding affinity (E_{S}) of nucleotide-free, ATP- or ADP.Pi-kinesin for MT.

S2. Potential characterizing the effect of NL docking of the MT-bound head on the movement of the tethered ADP-head

In our model, the NL docking of the MT-bound head provides an energy barrier E_{NL} to prevent the tethered ADP-head from moving backward but allow the head to move forward freely. Thus, the effect of the NL docking on the motion of the tethered ADP-head relative to the MT-bound head can be approximately characterized by a

potential $V_{NL}(x)$ having the form

$$V_{NL}(x) = E_{NL}, \qquad x \le 0, \tag{S1}$$

$$V_{NL}(x) = -E_{NL}(x-1), \qquad 0 < x \le 1 \text{ nm}, \tag{S2}$$

$$V_{NL}(x) = 0, \qquad x > 1 \text{ nm}, \tag{S3}$$

where the MT-bound head is located at $(x, y, z) = (0, 0, 0)$.

S3. Potential of interaction between two kinesin heads

We take the potential of interaction between the MT-bound head and detached ADP-head with the NL of the MT-bound head being undocked having following form

$$V_I(x,y,z,\alpha,\theta,\phi) = -E_{I1}\exp\left(-\frac{\sqrt{(x-x_1)^2+(y-y_1)^2+(z-z_1)^2}}{A_r}\right) \times\exp\left(-\frac{|\alpha-\alpha_1|}{A_\alpha}\right)\exp\left(-\frac{|\theta-\theta_1|}{A_\theta}\right)\exp\left(-\frac{|\phi-\phi_1|}{A_\phi}\right), \tag{S4}$$

where (x, y, z) is the center-of-mass coordinate of the detached ADP-head relative to that of the MT-bound head (which is taken as the origin of the coordinate) during one stepping period, (x_1, y_1, z_1) is the position of the detached ADP-head in the intermediate state, α_1, θ_1 and ϕ_1 are the nutation, rotation and precession angles of the detached ADP-head in the intermediate state, $E_{I1} > 0$ is the strong interaction strength and $A_r = 1$ nm characterizes the interaction distance. Based on the available structural data [S2], we take $\alpha_1 = 180°$, $\theta_1 = -80°$ and $\phi_1 = 0$ in the calculation, thus giving $(x_1, y_1, z_1) = 2r_{head}\times(\cos 80°, \sin 80°, 0)$. We have checked that taking other values of α_1, θ_1 and ϕ_1 has nearly no effect on our results. When the NL of the MT-bound head is docked, the potential is still described by Eq. (S4), but with E_{I1} being replaced with E_{I2} ($< E_{I1}$).

S4. Interaction between the NL and kinesin head

To determine the interaction between the NL and kinesin head, we should determine the elasticity of the linker by using all-atom MD simulations (see Section S8), as used elsewhere [S3–S5]. Here, we take the NL of wild-type *Drosophila* kinesin-1 as an example to describe the simulation procedure. We take residues 324 through 338 from the structural data (PDB 3KIN), where residues 325 – 338 constitute the NL. We adjust the line connecting the alpha carbon (CA) atom of

residue 324 and that of residue 338 along a given direction. We fix the CA atom of the residue 324 and impose a series of constant forces on the CA atom of the residue 338 along the given direction, as done before [S6]. We then calculate the distance, r_{NL}, between the two terminal CAs of the NL after reaching the equilibrium for 20 ns. In the literature, the calculated data of the force-extension relation of a flexible peptide were usually fitted by using the worm-like-chain (WLC) model. However, it is noted that the simulated data of the force-extension relation of the kinesin's NL are better fitted by using the exponential function than WLC model, especially at small values of pulling force (see Fig. S2). Thus, here we use the exponential function to fit the simulated data

$$F_{\mathrm{NL}}\left(r_{\mathrm{NL}}\right) = a\exp\left(br_{\mathrm{NL}}\right), \tag{S5}$$

where a and b are constants. Then the force on the detached head, which results from the stretched NL, can be calculated by using Eq. (S5).

Similarly, we obtain the force-extension relation of the NL of kinesin-3 head. The simulated data for kinesin-3's NL can also be fitted well using Eq. (S5) (Fig. S3).

S5. Equations to describe the movement of tethered ADP-head relative to nucleotide-free, ATP- or ADP.Pi-head bound fixedly to MT

We define the coordinate *oxyz* as shown in Fig. S1a, where the origin of the coordinate (0,0,0) is at the center-of-mass position of the MT-bound head. We consider the translation motion of the ADP-head in three dimensions (denoted by coordinates x, y and z) and rotation in three directions. The rotation is described by nutation motion (characterized by angle α), rotation motion (characterized by angle θ) and precession motion (characterized by angle ϕ). When the kinesin head is in the state bound to MT, α, θ and ϕ correspond to the angles of rotation in *xoz*, *xoy* and *yoz* planes, respectively. The translation and rotation of the ADP-head relative to the MT-bound head in viscous solution can be described by Langevin equations

$$\Gamma_x \frac{\partial x}{\partial t} = -\frac{\partial V_{\mathrm{W}}(x,y,z,\alpha,\theta,\phi)}{\partial x} - \frac{\partial V_{\mathrm{I}}(x,y,z,\alpha,\theta,\phi)}{\partial x} - F_{\mathrm{NL}}\left(\frac{r}{2}\right)\frac{x}{r} - \frac{\partial V_{\mathrm{NL}}(x)}{\partial x} + \xi_x(t), \tag{S6}$$

$$\Gamma_y \frac{\partial y}{\partial t} = -\frac{\partial V_{\mathrm{W}}(x,y,z,\alpha,\theta,\phi)}{\partial y} - \frac{\partial V_{\mathrm{I}}(x,y,z,\alpha,\theta,\phi)}{\partial y} - F_{\mathrm{NL}}\left(\frac{r}{2}\right)\frac{y}{r} + \xi_y(t), \tag{S7}$$

$$\Gamma_z \frac{\partial z}{\partial t} = -\frac{\partial V_{\mathrm{W}}(x,y,z,\alpha,\theta,\phi)}{\partial z} - \frac{\partial V_{\mathrm{I}}(x,y,z,\alpha,\theta,\phi)}{\partial z} - F_{\mathrm{NL}}\left(\frac{r}{2}\right)\frac{z}{r} + \xi_z(t), \tag{S8}$$

$$\Gamma_\alpha \frac{\partial \alpha}{\partial t} = -\frac{\partial V_W(x,y,z,\alpha,\theta,\phi)}{\partial \alpha} - \frac{\partial V_I(x,y,z,\alpha,\theta,\phi)}{\partial \alpha} + \xi_\alpha(t), \tag{S9}$$

$$\Gamma_\theta \frac{\partial \theta}{\partial t} = -\frac{\partial V_W(x,y,z,\alpha,\theta,\phi)}{\partial \theta} - \frac{\partial V_I(x,y,z,\alpha,\theta,\phi)}{\partial \theta} + \xi_\theta(t), \tag{S10}$$

$$\Gamma_\phi \frac{\partial \phi}{\partial t} = -\frac{\partial V_W(x,y,z,\alpha,\theta,\phi)}{\partial \phi} - \frac{\partial V_I(x,y,z,\alpha,\theta,\phi)}{\partial \phi} + \xi_\phi(t), \tag{S11}$$

where $r = \left(x^2 + y^2 + z^2\right)^{1/2}$, $V_W(x,y,z,\alpha,\theta,\phi)$ is the potential of the ADP-head interacting with the binding site on MT during the period after Pi release and before ADP release with the other nucleotide-free or ATP- or ADP.Pi-head binding fixedly to MT (see Section S1), $V_{NL}(x)$ is the potential characterizing the effect of the NL docking to the MT-bound head on the motion of the detached ADP-head (see Section S2), $V_I(x,y,z,\alpha,\theta,\phi)$ is the potential of interaction between the two kinesin heads (see Section S3), F_{NL} is the force acting on the ADP-head that results from the stretching of the NLs (see Section S4). The drag coefficients on the kinesin head are $\Gamma_x = \Gamma_y = \Gamma_z = 6\pi\eta_0 r_{head}$ and $\Gamma_\alpha = \Gamma_\theta = \Gamma_\phi = 8\pi\eta_0 r_{head}^3$, where η_0 is the solution viscosity in the vicinity of MT. Since the viscosity in the vicinity of MT is larger than that far away from MT, we take $\eta_0 = 0.02$ g cm^{-1} s^{-1} that is about 2-fold larger than that in water. Term $\xi_i(t)$ $(i = x, y, z, \alpha, \theta, \phi)$ is the fluctuating Langevin force acting on the kinesin head, with $\langle \xi_i(t) \rangle = 0$, $\langle \xi_i(t)\xi_j(t') \rangle = 0$ $(i \neq j)$ and $\langle \xi_i(t)\xi_i(t') \rangle = 2k_B T \Gamma_i \delta(t - t')$, where $k_B T$ is the thermal energy. Due to the steric restriction of MT and considering the size of the kinesin head with radius $r_{head} = 2.5$ nm, it is required that $y \geq y_0 = 0$ and $r \geq 2r_{head} = 5$ nm. The initial conditions for Eqs. (S6) – (S11) are: $(x_0, y_0, z_0, \alpha_0, \theta_0, \phi_0) = (-d, 0, 0, 0, 0, 0)$, with $d = 8.2$ nm being the periodicity of an MT filament.

S6. Equations to describe the movement of kinesin dimer in the intermediate state with one ADP-head bound to MT and the other ADP-head bound strongly to the MT-bound head

When the two ADP-heads are bound together strongly, the movement of the MT-bound ADP-head relative to MT can be described by following equations

$$\Gamma_x \frac{\partial x}{\partial t} = -\frac{\partial V_W(x,y,z,\alpha,\theta,\phi)}{\partial x} + \xi_x(t), \tag{S12}$$

$$\Gamma_y \frac{\partial y}{\partial t} = -\frac{\partial V_W(x,y,z,\alpha,\theta,\phi)}{\partial y} + \xi_y(t), \tag{S13}$$

$$\Gamma_z \frac{\partial z}{\partial t} = -\frac{\partial V_W(x,y,z,\alpha,\theta,\phi)}{\partial z} + \xi_z(t), \tag{S14}$$

$$\Gamma_\alpha \frac{\partial \alpha}{\partial t} = -\frac{\partial V_W(x,y,z,\alpha,\theta,\phi)}{\partial \alpha} + \xi_\alpha(t), \tag{S15}$$

$$\Gamma_\theta \frac{\partial \theta}{\partial t} = -\frac{\partial V_W(x,y,z,\alpha,\theta,\phi)}{\partial \theta} + \xi_\theta(t), \tag{S16}$$

$$\Gamma_\phi \frac{\partial \phi}{\partial t} = -\frac{\partial V_W(x,y,z,\alpha,\theta,\phi)}{\partial \phi} + \xi_\phi(t). \tag{S17}$$

The initial conditions for Eqs. (S12) – (S17) are: ($x_0, y_0, z_0, \alpha_0, \theta_0, \phi_0$) = (0,0,0,0,0,0).

S7. Monte-Carlo simulations

In the Monte-Carlo simulations, during each time step Δt ($\Delta t = 10^{-4}$ s in our simulation) a random number *ran* is generated with uniform probability between 0 and 1. The state transition with rate constant k_i (i = T, c, NL, D) takes place if *ran* ≤ P_i and the transition does not take place if *ran* > P_i. Here, $P_i = k_i \Delta t$ is the probability of state transition in each time step Δt, $k_T = k_b$[ATP] represents ATP-binding rate to the nucleotide-free head, with k_b being the second-order rate constant for ATP binding and [ATP] being the ATP concentration, k_c represents the rate of ATP hydrolysis and Pi release, k_{NL} represents the rate constant of NL docking into the motor domain of MT-bound head in ATP or ADP.Pi state when the detached ADP-head is in the intermediate position, and k_D represents the rate constant of ADP releasing from ADP-head.

S8. All-atom MD simulations

The all-atom MD simulations are carried out by using GROMACS5.1 [S7] with OPLS-AA/L all-atom force field [S8]. To avoid the edge effect, the distance between the peptide of the NL and the boundary of the box is at least 1.5 nm and much longer along the direction of the pulling force to stretch the NL. We add solvent and necessary ions with favorable concentration. Counter-ions are also added to neutralize the system. All MD simulations are run at 300K and 1 bar. The time step is set as 2 fs, and the output data is updated every ps. All chemical bonds are constrained using LINCS algorithm [S9]. The short-range electrostatics interaction and the cutoff for

van der Waals interaction is set as 1 nm. Velocity-rescaling temperature coupling [S10] and Berendsen pressure coupling [S11] are used. The energy minimization is performed using the steepest descent method. Before the dynamic simulations, the systems are equilibrated successfully for 2 ns at 300 K and 1 bar pressure in the NVT ensemble and NPT ensemble, respectively. For calculations of the force-extension relation of the NL, after a 20-ns constant force-extension simulation, the distance between the two terminal CAs of the linker is extracted from the output trace files using the VMD1.9.2 [12].

References

[S1] P. Xie, S.-X. Dou and P.-Y. Wang, Processivity of single-headed kinesin motors. *Biochimica et Biophysica Acta (BBA) – Bioenergetics*, 2007, **1767**, 1418–1427

[S2] M. C. Alonso, D. R. Drummond, S. Kain, J. Hoeng, L. Amos and R. A. Cross, An ATP gate controls tubulin binding by the tethered head of kinesin-1. *Science*, 2007, **316**, 120-123.

[S3] Z.-W. Duan, P. Xie, W. Li and P.-Y. Wang, Are coiled-coils of dimeric kinesins unwound during their walking on microtubule? *PLOS ONE*, 2012, **7**, e36071.

[S4] Y.-B. Fu, Z.-F. Wang, P.-Y. Wang, P. Xie, Optimal numbers of residues in linkers of DNA polymerase I, T7 primase and DNA polymerase IV. *Scientific Reports*, 2016, **6**, 29125.

[S5] Y.-B. Fu, Y.-R. Liu, P.-Y Wang, P. Xie, A revised worm-Like chain model for elasticity of polypeptide chains. *Journal of Polymer Science, Part B: Polymer Physics*, 2018, **56**, 297–307.

[S6] V. Hariharan and W. O. Hancock, Insights into the mechanical properties of the kinesin neck linker domain from sequence analysis and molecular dynamics simulations. *Cellular and Molecular Bioengineering*, 2009, **2**, 177-189.

[S7] M. J. Abraham, T. Murtola, R. Schulz, S. Páll, J. C. Smith, B. Hess and E. Lindahl, GROMACS: high performance molecular simulations through multi-level parallelism from laptops to supercomputers. *SoftwareX*, 2015, **1–2**, 19-25.

[S8] W. L. Jorgensen, D. S. Maxwell and J. Tirado-Rives, Development and testing of the OPLS all-atom force field on conformational energetics and properties of organic liquids. *Journal of the American Chemical Society*, 1996, **118**, 11225-11236.

[S9] B. Hess, P-LINCS: A parallel linear constraint solver for molecular simulation. *Journal of Chemical Theory and Computation*, 2008, **4**, 116-122.

[S10] G. Bussi, D. Donadio and M. Parrinello, Canonical sampling through velocity rescaling. *The Journal of Chemical Physics*, 2007, **126**, 014101.

[S11] H. J. C. Berendsen, J. P. M. Postma, W. F. van Gunsteren, A. DiNola and J. R. Haak, Molecular dynamics with coupling to an external bath. *The Journal of Chemical Physics*, 1984, **81**, 3684-3690.

[S12] W. Humphrey, A. Dalke and K. Schulten, VMD - visual molecular dynamics. *Journal of Molecular Graphics*, 1996, **14**, 33-38.

SI figures

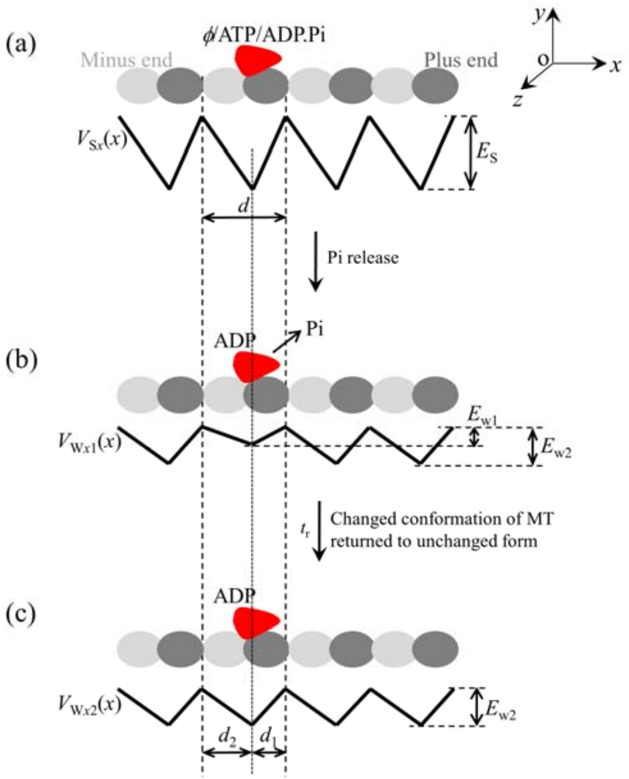

Fig. S1. Interaction potentials between a kinesin head and MT in the *x* direction (along an MT protofilament) during one ATPase cycle. **(a)** Strong interaction potential, $V_{Sx}(x)$, in nucleotide-free, ATP or ADP.Pi states. The top figure shows the position of the kinesin head relative to the MT. **(b)** Weak interaction potential, $V_{Wx1}(x)$, in ADP state immediately after Pi release. The top figure shows the position of the kinesin head relative to the MT. **(c)** Weak interaction potential, $V_{Wx2}(x)$, in ADP state in a period of time t_r after Pi release. The top figure shows the position of the kinesin head relative to the MT.

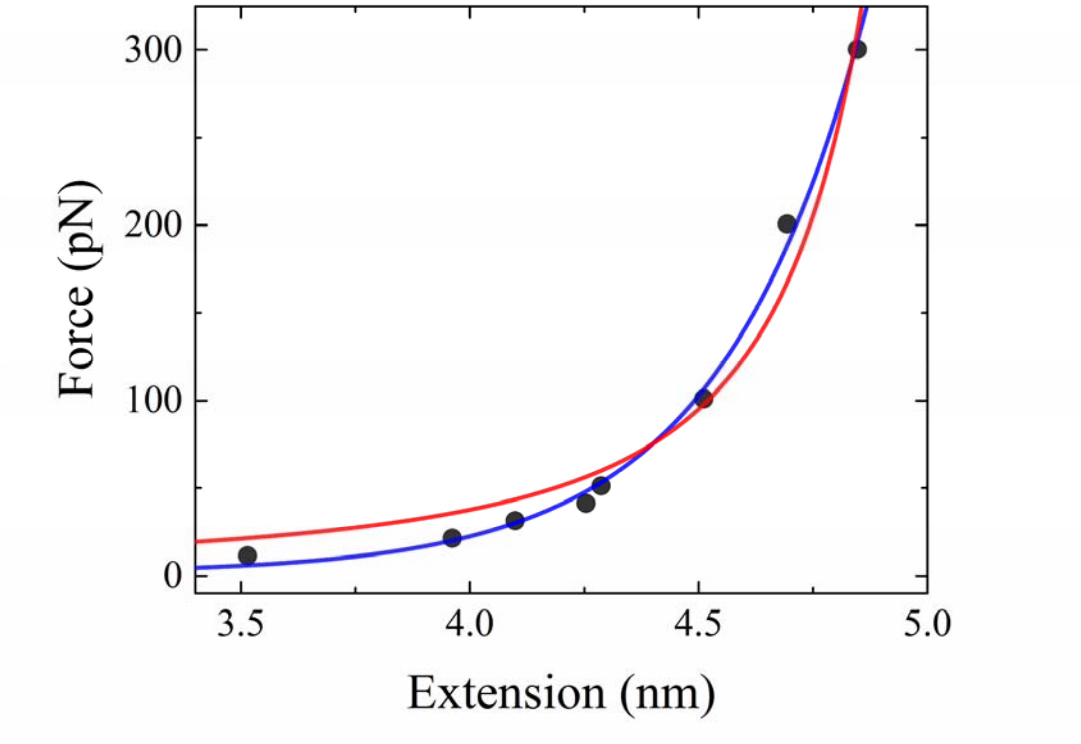

Fig. S2. Force-extension relation of the NL of *Drosophila* kinesin-1 head. Dots represent the results obtained by using MD simulations. Red lines is the fit curve with WLC model, $f(x) = k_B T / (4L_p) \left[(1 - x/L_c)^{-2} - 1 + 4x/L_c \right]$, where the contour length L_c = 5.26 nm and the persistence length L_p = 0.56 nm. Blue line is the fit curve with Eq. (S5), $F_{NL}(r_{NL}) = a \exp(b r_{NL})$, with $a = 7.064 \times 10^{-5}$ pN and b = 3.148 nm^{-1}.

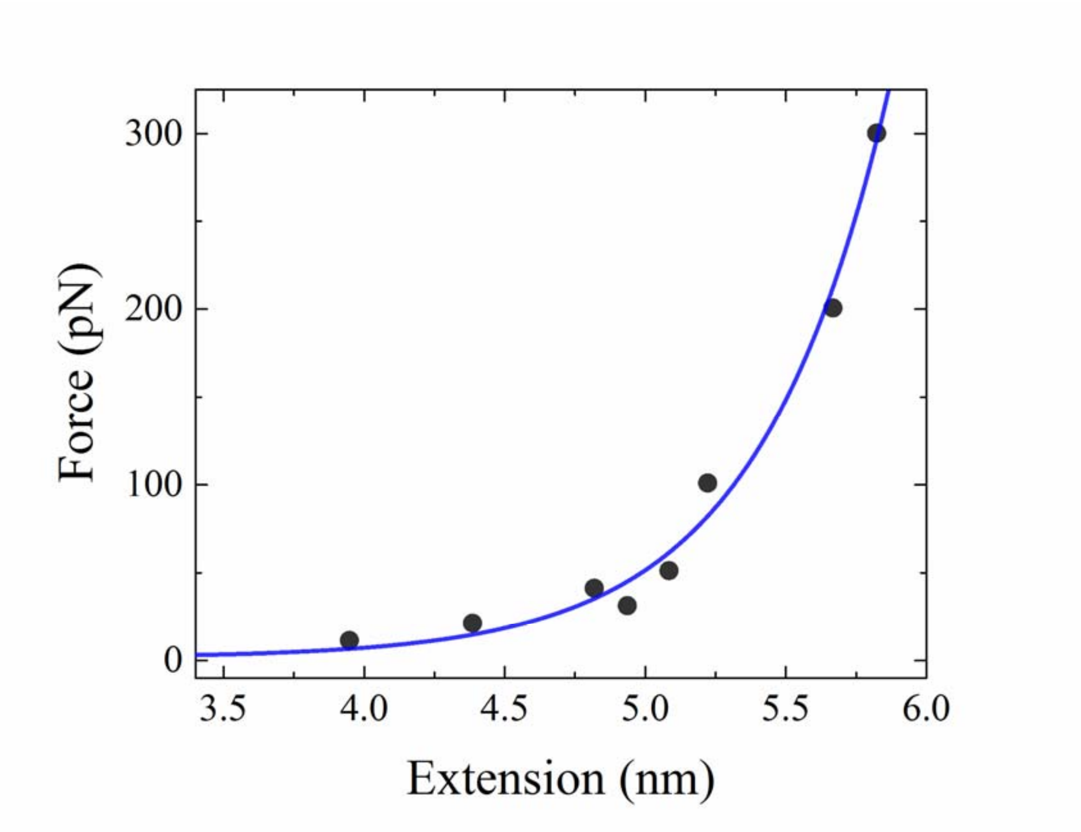

Fig. S3. Force-extension relation of the NL of kinesin-3 head. Dots represent the results obtained by using all-atom MD simulations. Line is the fit curve with Eq. (S5), with $a = 1.060 \times 10^{-3}$ pN and $b = 2.150$ nm^{-1}.
